# Reactive Balance in Adolescent Idiopathic Scoliosis: A Prospective Motion Analysis Study

**DOI:** 10.3390/jcm14051715

**Published:** 2025-03-04

**Authors:** Ria Paradkar, Christina Regan, Kathie Bernhardt, Kenton R. Kaufman, Todd A. Milbrandt, A. Noelle Larson

**Affiliations:** Department of Orthopedic Surgery, Mayo Clinic, Rochester, MN 55905, USA; paradkar.ria@mayo.edu (R.P.); regan.christina@mayo.edu (C.R.); bernhardt.kathie@mayo.edu (K.B.); kaufman.kenton@mayo.edu (K.R.K.); milbrandt.todd@mayo.edu (T.A.M.)

**Keywords:** adolescent idiopathic scoliosis, reactive balance, vertebral body tethering, spinal fusion, gait stability

## Abstract

**Background/Objectives**: Traditional fusion leads to a loss of spine mobility across the fused vertebrae. Vertebral body tethering (VBT) was developed with the goal of increasing flexibility and maintaining some spinal mobility. However, it is not known if the additional mobility leads to significant functional improvement. This prospective motion analysis study evaluates functional outcomes, specifically gait stability, in pre-operative, post-fusion, and post-VBT patients by using postural perturbations on a treadmill. **Methods**: Overall, 79 subjects underwent a computer-controlled treadmill study with postural perturbations, which simulated trips and slips. The subjects were harnessed for safety. Overall, 21 subjects were healthy controls, 18 patients were at least one-year post-VBT, 15 patients were at least one-year post-fusion, and 25 were pre-operative scoliosis patients. Subject weight, height, and treadmill acceleration were recorded and used to determine anteroposterior single (ASSTs, PSSTs) and multiple (AMSTs, PMSTs) stepping thresholds to describe the maximum torque a patient could withstand before failing to recover from the simulated trip. Independent t-tests were run to compare groups under the advice of a master statistician with expertise in orthopedic surgery. **Results**: Pre-operative scoliosis patients had lower PSSTs than healthy controls (uncorrected *p* = 0.036). No significant differences were observed between pre-operative and post-operative groups for both fusion and VBT. There was no significant difference in ASST, AMST, or PMST between any of the groups. **Conclusions**: The lower PSST in pre-operative scoliosis patients compared to healthy controls may reflect impaired reactive balance and potentially increased fall risk. Interestingly, there was no significant difference in reactive balance measures between pre-operative and post-operative scoliosis patients or between post-fusion and post-VBT patients.

## 1. Introduction

Adolescent idiopathic scoliosis (AIS) is a spinal deformity, characterized by lateral curvature of the spine, that affects 1–3% of adolescents [1]. This condition not only impacts physical appearance, but can also lead to functional limitations and psychosocial effects. Symptoms of AIS include chest deformity, trunk asymmetry, and pain, which can adversely affect activity level, self-esteem, and social interactions in adolescents [2,3]. The management of scoliosis is guided by the Cobb angle. Mild curves are generally monitored or managed non-operatively with bracing. In contrast, more severe cases with Cobb angles greater than 50 degrees often require surgical treatment.

The traditional surgical treatment for severe scoliosis is spinal fusion, which involves fusing vertebrae to correct the deformity and stabilize the spine [4]. While spinal fusion is effective for curve correction and halting curve progression, it is associated with the restriction of spinal mobility and flexibility [5]. By eliminating spinal motion at the fused segments, spinal fusion can impact functional activities, especially those requiring bending or twisting. Adolescents who have undergone spinal fusion report increased perception of stiffness following surgery, which can affect their ability to perform everyday tasks [6]. These limitations are particularly concerning for active children and young adults in which physical restrictions can reduce participation in sports and extracurricular activities, potentially contributing to feelings of isolation and impacting quality of life during fundamental periods of development.

To address these limitations, vertebral body tethering (VBT) has emerged as an alternative to spinal fusion that aims to preserve growth and motion [7,8]. Unlike fusion, VBT allows for the dynamic correction of scoliosis by applying tension via a flexible cord anchored to the vertebral bodies. Current evidence suggests that VBT may preserve lumbar mobility in the post-operative lumbar spine as well as anterior and lateral flexibility [9,10,11]. Additionally, patients who underwent VBT reported improved quality of life and satisfaction, as measured by higher SRS-22 (a scoliosis-specific outcome measure that assesses function, pain, self-image, mental health, and satisfaction with treatment) and SF-36 (a general health survey that evaluates physical and mental health) scores, underscoring its potential advantage over traditional fusion. Despite these findings, it remains unclear whether these biomechanical differences confer any functional benefits.

To date, much of the focus of scoliosis management has revolved around preventing curve progression and correcting the spinal deformity. However, there is increasing recognition of the importance of patient-centered outcomes and research into the link between structural alignment and real-world functionality. Gait stability, balance, and flexibility are critical components of functional mobility. Studies have shown that scoliosis affects balance and alters gait patterns [12,13,14,15]. These deficits may be exacerbated in severe cases of scoliosis with significant spinal asymmetry. Research indicates that patients with AIS display abnormal movement patterns and muscle activation during many activities of daily living, including level walking and climbing stairs [16]. While many children with scoliosis remain active and participate in sports, it is possible that compromised balance and gait mechanics increase the risk of pain and susceptibility to injury. Examining postural balance in children with scoliosis is important to better understand the functional challenges associated with scoliosis and interventions that can address them.

Previous investigations have employed various methods to assess static and dynamic balance [17]. However, static balance may not completely capture the dynamic challenges encountered in real-life activities. Dynamic balance, which refers to the ability to maintain stability after an external disturbance, involves precise adjustments to prevent falling [18]. This requires combined efforts from the sensory and musculoskeletal systems to integrate visual and proprioceptive input and perform corrective movements to counteract the external force applied. Dynamic balance is crucial for everyday activities like walking, climbing stairs, and participating in sports. Nearly all neuromusculoskeletal disorders, including scoliosis, lead to the degeneration of balance control mechanisms. Accordingly, assessing reactive balance in scoliosis patients is a critical part of evaluating overall functional mobility.

Treadmill-induced perturbations, which simulate slips and trips, offer a novel approach to evaluate dynamic balance. Treadmill-induced perturbations measure an individual’s ability to recover from unexpected treadmill belt translocations. Successful recovery from surface translations requires a coordinated response with a quick reaction time, sufficient joint movement, and control of the center of mass [19]. Treadmill-induced perturbations have been employed to evaluate gait stability in various populations, including older adults and patients with neurological conditions [20,21]. A study by Crenshaw et al. demonstrated that posterior stepping thresholds, measured via treadmill perturbations, predict fall risk in elderly adults [22]. However, this methodology has not been applied to scoliosis patients, particularly in the context of post-surgical changes.

This prospective motion analysis study aims to investigate gait stability and postural control in pre-operative and post-operative patients with scoliosis. This study is the first to use treadmill-induced perturbations to quantify reactive balance in patients with AIS. By assessing anteroposterior stepping thresholds, we aim to determine whether the surgical treatment of scoliosis by spinal fusion or VBT affects functional stability. The findings of this study will contribute to the understanding of how different treatment modalities influence balance and mobility in adolescents with scoliosis, providing valuable insights for clinical practice.

## 2. Materials and Methods

This study was a prospective cohort study. Institutional review board approval was obtained prior to the initiation of the study (15-005602).

### 2.1. Participants

A total of 79 subjects participated in the study. The cohort consisted of 21 healthy control patients, 15 patients who were at least one-year post-spinal fusion, 18 patients who were at least one-year post-VBT, and 25 pre-operative scoliosis patients. Participants were recruited from a single institution’s pediatric orthopedic clinic. Patients under the age of 18 who were diagnosed with AIS met inclusion criteria for this study. Exclusion criteria included non-idiopathic etiologies of scoliosis and an inability to participate in the treadmill-induced perturbation protocol due to comorbidities. All participants and their guardians provided informed consent prior to enrolling in this study.

### 2.2. Procedures

**Figure 1:**
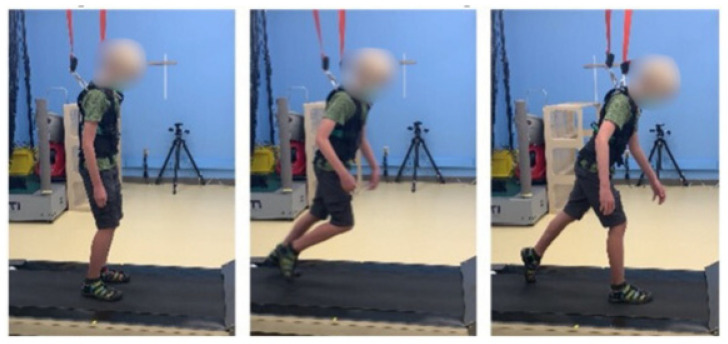
Computer-controlled treadmill simulation setup with patient in harness.

Anteroposterior stepping thresholds were assessed using a computer-controlled treadmill (ActiveStep^®^, Simbex, Lebanon, NH, USA) that delivered postural perturbations to simulate slips and trips. Participants stood on the treadmill in their own closed-toe shoes and were attached to a harness connected to an overhead rail for safety (Figure 1). The harness was adjusted to prevent hand or knee contact with the treadmill in case of a fall during the perturbation trials. A brief orientation to the treadmill setup was given to familiarize participants with the equipment prior to the trials. All perturbation trials were conducted in a controlled environment with standardized lighting and noise levels to minimize external distractions.

Once patients were appropriately harnessed, two series of perturbations were applied. One series of treadmill perturbations quantified anterior and posterior single-stepping thresholds (ASSTs and PSSTs) in which participants were instructed to “try not to step”. The second series of treadmill perturbations quantified anterior and posterior multiple-stepping thresholds (AMSTs and PMSTs) in which participants were instructed to “try to take only one step”. In order to prevent anticipatory improvements in stepping-thresholds, the direction and timing of perturbations was pseudo-randomized using a custom software macro (Excel 2010, Microsoft Corporation, Redmond, WA, USA). For both series, perturbation directions were randomized, with no more than three consecutive perturbations delivered in the same direction. Following participant acknowledgement of readiness, perturbations were triggered after a randomized delay (time between the initiation of disturbance by the investigator and when the treadmill belt moved) of 3–10 s. If a participant failed to comply with stepping instructions in four consecutive trials, the corresponding perturbation magnitude was considered a threshold. Failures were defined as follows: (i) stepping in response to the perturbation when instructed not to, (ii) taking more steps than instructed, or (iii) relying on the harness.

The well-established inverted pendulum model was used as the biomechanical basis to analyze reactive balance. This model describes the body as an upside-down pendulum with the feet representing the pivot point and the rest of the body swaying in response to movement or external forces [18]. The perturbation magnitude at the stepping threshold was recorded and expressed as torque (τ = |m·a·l|), where m is body mass, a is the perturbation acceleration, and l is the estimated pendulum height (0.586 · height), representing the destabilizing torque at the base of the inverted pendulum.

### 2.3. Statistical Analysis

All stepping thresholds were normalized to unitless values in order to account for differences in height and body mass. This was carried out by dividing raw stepping thresholds by (m·g·l), where m is body mass in kilograms, g is 9.81 m/s^2^, and l is trochanteric height in centimeters, which was estimated as 48.5% of total body height.

In order to identify differences in reactive balance by surgical status and type of scoliosis surgery, participants were categorized into the following groups: control, pre-op scoliosis, post-fusion, and post-tether.

To evaluate differences in reactive balance, we tested the following predefined hypotheses:

1. Control vs. pre-op: pre-op scoliosis patients will have reduced stepping thresholds compared to healthy controls, indicating impaired reactive balance in scoliosis patients.
2. Pre-op vs. post-op: post-op patients will have improved stepping thresholds compared to their pre-op counterparts, supporting the effectiveness of fusion surgery in restoring reactive balance.
3. Post-fusion vs. post-tether: post-tether patients will have improved stepping thresholds compared to post-fusion patients, indicating that VBT may have functional benefits compared to fusion surgery.

Independent two-sample *t*-tests were used to evaluate these predefined hypotheses. This approach allowed us to focus our analysis on clinically meaningful comparisons which align with the study’s objectives. Additionally, Spearman correlation coefficients were utilized to assess the relationship between the number of levels fused or the number of levels tethered and normalized stepping thresholds. A *p*-value of less than 0.05 was considered statistically significant. All analyses were performed using BlueSky Statistics 10.3.4 (BlueSky Statistics LLC.; Chicago, IL, USA).

## 3. Results

### Patient Demographics

A total of 79 subjects underwent postural perturbation to assess reactive balance. Participants were categorized into control (n = 21), pre-op (n = 24), post-fusion (n = 15), and post-tether (n = 18) groups. Participant demographics, including age, height, weight, BMI, major Cobb angle, number of levels instrumented, and gender, are summarized in Table 1.

Reactive balance measures, including the anterior single-stepping threshold (ASST), the posterior single-stepping threshold (PSST), the anterior multiple-stepping threshold (AMST), and the posterior multiple-stepping threshold (PMST) for each group, are shown in Table 2. The pre-op group consisted of patients who were scheduled to undergo fusion or VBT and the post-op group consisted of patients who were at least one-year post-fusion or post-tether.

**Figure 2:**
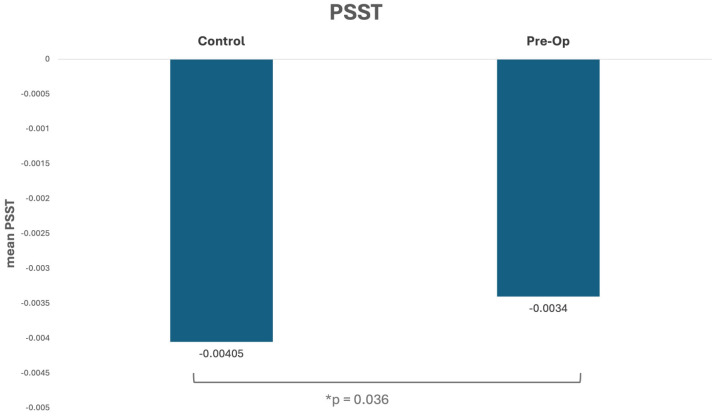
Control vs. pre-op scoliosis posterior single-stepping threshold.

The results showed a significant difference in PSST between the control group and the pre-op scoliosis group, as demonstrated in Figure 2. The control group had a higher mean PSST (0.00405, SD = 0.0010) compared to the pre-op group (0.00340, SD = 0.0010), which indicates that pre-op scoliosis patients may have impaired balance compared to healthy controls (*p* = 0.0366). Similar to previous work in adults in our lab, no significant differences were found between groups for ASST, AMST, or PMST.

For the post-fusion and post-tether groups, Spearman correlation coefficients were determined to assess the relationship between the number of levels fused or number of levels tethered and normalized stepping thresholds (ASST, PSST, AMST, PMST) (Table 3). Only one correlation coefficient was significant, showing a weakly positive correlation between the number of levels fused and the PSST (r_s_ = 0.520, *p* = 0.047). All other relationships were weak and not statistically significant.

## 4. Discussion

The purpose of this study was to assess whether scoliosis contributes to imbalance and to determine if surgical interventions such as spinal fusion and VBT confer any functional benefit. The findings of this study demonstrate that scoliosis may be associated with impaired reactive balance, as evidenced by the lower PSST in pre-op scoliosis patients compared to healthy controls. Additionally, this study did not detect any significant difference in reactive balance measures between pre-operative and post-operative patients, suggesting that in this cohort, neither spinal fusion nor VBT significantly improved balance. These findings suggest the need for interventions that address impaired balance beyond the surgical correction of the spinal deformity. 

The observed difference in PSST between pre-operative scoliosis patients and the control group may support the hypothesis that scoliosis contributes to the impairment of balance. PSST is a measure of reactive balance that quantifies a participant’s ability to recover from posterior postural perturbation without taking any steps. A lower PSST could indicate compromised gait stability in patients with scoliosis. This is especially important to consider because of the critical development in neuromuscular and musculoskeletal growth that occurs in adolescence. Impaired gait stability may have implications in everyday activities, especially in situations that require rapid recovery from sudden shifts in body position, such as regaining balance after trips or slips, navigating uneven terrain, or participating in sports. Various interrelated factors may contribute to the imbalance observed in scoliosis including spinal asymmetry, altered spinal mechanics, irregular muscle recruitment, and altered proprioception.

Scoliosis is a three-dimensional vertebral malalignment that has been shown to affect both gait and balance [23]. Previous studies have found that patients with AIS have an altered center of mass and center of pressure [24,25]. In addition to altered spinal mechanics, AIS is also associated with muscular imbalance. Paravertebral muscles are crucial for maintaining an upright position. Studies show that patients with scoliosis have larger paravertebral cross-sectional areas on the concave side of the deformity compared to the convex side [26,27]. Additionally, deficits in integration of input from vestibular, visual, and proprioceptive pathways have been observed in AIS patients, potentially contributing to postural instability and impaired balance [28,29]. Collectively, these studies show that structural, neuromuscular, and sensory changes could lead to deficits in balance in scoliosis patients.

The reduced PSST observed in scoliosis patients in this study aligns with previous research. Notably, a study by Crenshaw et al. found that PSSTs were predictive of falls in older women, underscoring the clinical relevance of this measure [22]. The decreased PSST in pre-op scoliosis patients may suggest a similarly increased fall risk. For adolescents with AIS, this reduced stability may affect participation in physical activities, increase the likelihood of injury, and impact quality of life.

Interestingly, no significant changes in reactive balance measures were observed between pre-op and post-op patients. Patients in the pre-op group had an average major Cobb angle of 53 degrees compared to 21 degrees and 28 degrees in the post-fusion and post-tether groups, respectively. This suggests that in this limited cohort, despite the correction of spinal curvature, surgical intervention may not address underlying balance deficits in patients with scoliosis. The analysis of the relationship between the number of levels instrumented in post-fusion and post-tether groups and all normalized stepping thresholds revealed only one significant relationship—a weakly positive correlation between the number of levels fused and ASST (r_s_ = 0.520, *p* = 0.047). However, no significant correlations were found between the number of levels fused and ASST, AMST, and PMST or between the number of levels tethered and any of the stepping thresholds. This suggests that the number of levels instrumented may not play a critical role in reactive balance; however, further studies should examine this with larger post-operative cohorts. In addition, there was no significant difference in balance following spinal fusion versus VBT. A study by Oeding et al. showed that VBT patients show a significantly quicker return to common adolescent activities, including participation in sports, running, and physical education (PE) classes, compared to fusion patients. VBT patients were also less likely to have to give up activities that they performed prior to surgery due to changes in bending abilities compared to fusion patients [30]. Despite the body of evidence showing preserved spinal mobility following VBT, the results of this study indicate that both spine fusion and VBT may yield comparable results regarding postural stability [10]. The absence of significant changes in patients at least one-year post-fusion or post-VBT could also suggest that the recovery of balance and stability following surgical treatment of scoliosis is a more gradual process. A study with a longer follow-up time may be required to observe post-operative functional improvement.

The results of this study highlight several clinical implications for the management of patients with AIS. While surgical intervention with spinal fusion or VBT may address the spinal curvature associated with scoliosis, these procedures alone may not be sufficient to improve potential deficits in balance associated with scoliosis. Post-operative rehabilitation programs and physical therapy focused on strength and balance could enhance functional outcomes in patients post-operatively. Scoliosis-specific physical therapy methods such as Schroth therapy, which focuses on muscle strengthening and postural alignment, could be supplemented with interventions that target balance and proprioception, such as hippotherapy and sensory integration therapy [31,32,33]. Novel techniques such as perturbation training and virtual reality training have also demonstrated potential to improve reactive balance in preliminary studies [34,35]. Integrating these approaches into standardized protocols for physical therapy and rehabilitation following surgery may help optimize recovery.

This study has multiple limitations, including sample size, post-operative follow-up duration, and the influence of external factors on balance assessments. The relatively small sample sizes may affect the generalizability of findings and reduce thr statistical power of the study. Future studies should be conducted with larger cohorts to validate results. Including braced patients in future studies could offer further insight into changes in reactive balance in non-surgical treatment groups.

Another important limitation of this study was the one-year duration of follow-up for post-op patients. The recovery of balance following surgery may be a gradual process that requires a longer assessment period to capture. Longer follow-up durations may allow for a more comprehensive evaluation of how post-operative outcomes evolve over time. Furthermore, assessing reactive balance measures at multiple timepoints following surgery may offer valuable information about the trajectory of balance recovery in post-op patients.

Due to limited patient numbers, it is possible that our study was underpowered to predict a difference between tether and fusion patients, and the study design was a cross-sectional study of both pre- and post-operative patients. A post hoc power analysis showed that 98 pre-op and 98 post-op VBT patients would be needed to detect a 0.0004 difference in PSST to achieve 80% power with an alpha of 0.05. A study of the same patients pre-operatively and post-operatively would provide improved statistical power, and not having this remains a limitation of this study. However, we did show a difference between pre-operative scoliosis patients and normal controls, which warrants further investigation. Additionally, external factors and environmental conditions such as fear of falling, nervousness, or the mastery effect could impact participant performance during balance assessments with treadmill-induced perturbations. This is especially important to consider given that all participants in this study were children.

Future research should evaluate additional functional outcomes, such as step activity monitors, to better understand deficits associated with scoliosis and how surgical interventions may affect these [36]. The treatment of scoliosis with spinal fusion or VBT may have differential effects on other aspects of functional mobility including gait, flexibility, and strength. The further exploration of other dynamic balance measures such as lateral-stepping thresholds could provide further insight into the postural control deficits associated with scoliosis. Tailored balance rehabilitation for both pre-op and post-op scoliosis patients is another valuable area of research. Altogether, exploring these outcomes may aid in clinical decision-making and the development of effective interventions for patients with AIS.

## 5. Conclusions

This study highlights a potential association between scoliosis and impaired balance, as suggested by the lower PSST values in pre-op scoliosis patients compared to healthy controls. Interestingly, in our limited sample size, neither spinal fusion nor VBT patients showed improved balance outcomes following surgery. The results of this study could support the concept of targeted balance training to improve function in patients with scoliosis, but continued research is needed to elucidate the impact of scoliosis and surgical interventions on functional mobility.

## Figures and Tables

**Table 1:** Baseline characteristics of 79 study participants.

Group	Average Age (Years) ± SD	Average Height (cm) ± SD	Average Weight (kg) ± SD	Average BMI (kg/m^2^) ± SD	Average Major Cobb Angle (°) ± SD	Average Number Levels Instrumented ± SD	Male: Female
Control (n = 21)	12.7 ± 2.2	159.7 ± 12.7	51.2 ± 12.7	19.9 ± 3.3	N/A	N/A	5:16
Pre-op (n = 25)	13.2 ± 2.3	160.8 ± 9.5	51.4 ± 9.7	19.8 ± 3.0	53 ± 8.6	N/A	5:20
Post-fusion (n = 15)	16.9 ± 2.5	168.4 ± 10.9	64.8 ± 17.3	22.5 ± 4.1	21 ± 8.7	10.0 ± 2.3 (range: 5–12)	4:11
Post-tether (n = 18)	14.0 ± 1.5	166.0 ± 8.9	60.2 ± 16.1	21.7 ± 4.8	28 ± 7.9	7.8 ± 1.9 (range: 5–12)	2:16

SD = standard deviation.

**Table 2:** Reactive balance measures.

	Control	Pre-Fusion	Pre-Tether	Pre-Op	Post-Fusion	Post-Tether	Post-Op
n	21	8	17	25	15	18	33
mean ASST (SD)	0.00507 (0.0007)	0.00531 (0.0006)	0.00518 (0.0009)	0.00522 (0.0008)	0.00521 (0.0006)	0.00489 (0.0009)	0.00504 (0.0008)
mean PSST (SD)	−0.00405 (0.0010)	−0.00331 (0.0009)	−0.00344 (0.0011)	−0.00340 (0.0010)	−0.00349 (0.0007)	−0.00376 (0.0010)	−0.00364 (0.0009)
mean AMST (SD)	0.01707 (0.0027)	0.01584 (0.0032)	0.01572 (0.0036)	0.01576 (0.0034)	0.01511 (0.0034)	0.01608 (0.0029)	0.01564 (0.0031)
mean AMST (SD)	−0.01126 (0.0027)	−0.01021 (0.0036)	−0.01007 (0.0036)	−0.00971 (0.0040)	−0.01035 (0.0024)	−0.01074 (0.0028)	−0.01056 (0.0026)

SD = standard deviation.

**Table 3:** Spearman correlation coefficients for number of levels fused/tethered and normalized stepping thresholds.

Stepping Threshold	Spearman Correlation Coefficient	*p*-Value
**Correlation between Number Levels Fused and Normalized Stepping Thresholds**		
ASST	0.041	0.885
PSST	0.520	0.047 *
AMST	−0.237	0.474
PMST	0.200	60.2 ± 16.1
**Correlation between Number Levels Tethered and Normalized Stepping Thresholds**		
ASST	0.389	0.111
PSST	0.394	0.105
AMST	−0.397	0.103
PMST	0.181	0.472

* = significant value.

## Data Availability

Data will be made available upon reasonable request.
